# Neural Stem Cell-Conditioned Medium Ameliorated Cerebral Ischemia-Reperfusion Injury in Rats

**DOI:** 10.1155/2018/4659159

**Published:** 2018-04-03

**Authors:** HongNa Yang, Cuilan Wang, Hui Chen, Lan Li, Shuang Ma, Hao Wang, YaRu Fu, Tingyu Qu

**Affiliations:** ^1^Department of Critical Care Medicine, Qilu Hospital of Shandong University, Shandong University, Jinan, Shandong 250012, China; ^2^Department of Neurology, Qilu Hospital of Shandong University, Shandong University, Jinan, Shandong 250012, China; ^3^Department of Psychiatry, University of Illinois College of Medicine, 1601 West Taylor Street, Chicago, IL 60612, USA; ^4^R & D of Cell and Tissue Bank, Qilu Stem Cell Engineering Company of Shandong Province, Jinan, Shandong 250000, China

## Abstract

**Introduction:**

Our previous study suggested that NSC-CM (neural stem cell-conditioned medium) inhibited cell apoptosis *in vitro*. In addition, many studies have shown that neurotrophic factors and microparticles secreted into a conditioned medium by NSCs had neuroprotective effects. Thus, we hypothesized that NSC-CM had the capacity of protecting against cerebral I/R injury.

**Methods:**

Adult male Sprague-Dawley rats receiving middle cerebral artery occlusion surgery as an animal model of cerebral I/R injury were randomly assigned to two groups: the control group and NSC-CM-treated group. 1.5 ml NSC-CM or PBS (phosphate buffer saline) was administrated slowly by tail vein at 3 h, 24 h, and 48 h after ischemia onset.

**Results:**

NSC-CM significantly ameliorated neurological defects and reduced cerebral infarct volume, accompanied by preserved mitochondrial ultrastructure. In addition, we also found that NSC-CM significantly inhibited cell apoptosis in the ischemic hemisphere via improving the expression of Bcl-2 (B-cell lymphoma-2).

**Conclusion:**

NSC-CM might be an alternative and effective therapeutic intervention for ischemic stroke.

## 1. Introduction

Ischemic stroke is considered to the major cause of disability and the second cause of death in the world [1]. To date, tissue plasminogen activator (tPA) is still the only effective treatment. But, narrow treatment window (4.5 h) and relative high risk of intracerebral hemorrhage limited the clinical application of tPA [1]. In addition, neuroprotective agents tested in patients were proved to fail in clinical trials because of side effects and/or low efficacy although all such agents were demonstrated to decrease neuronal cell death and infarct size in cell culture and animal stroke model, respectively [1]. Thus, we need to find new therapy strategy to treat ischemic stroke.

Recently, stem cell-based therapy, especially neural stem cell (NSC) therapy, gained more and more attracts in treating ischemic stroke. Previous studies proved that NSCs were able to promote neurological recovery via direct action (neuronal replacement) [2] and indirect bystander actions secreting BDNF [3] (brain-derived neurotrophic factor), inhibiting the process of inflammation and enhancing endoneurogenesis [4]). But, the original resource, low survival, and neuronal differentiation rates [5] as well as the potential tumor formation of NSCs [6] limited their clinical application. Thus, stem cell-based treatment is not an ideal therapeutic intervention for ischemic stroke. However, it has been reported that NSCs released many neurotrophic factors, such as BDNF [7], GDNF (glial cell line-derived neurotrophic factor), NT-3 (neurotrophin-3) [8], and other soluble factors, as well as microvesicles (MVs) [9] in a culture-conditioned medium. In addition, there are also unknown neurotrophic factors and nondegraded mitotic factors, such as: bFGF (basic fibroblast growth factor) and EGF (epidermal growth factor). Especially, low circulating concentration of BDNF is associated with poor long-term functional outcome of ischemic stroke [10] while intranasal BDNF protected against cerebral I/R injury [11]. In addition, GDNF and NT-3 have been proved to exert neuroprotection against cerebral I/R injury [12, 13].

MVs (microvesicles) are nanosized, membrane-bound vesicles released from cells that can transport cargo—including DNA, RNA, and proteins—between cells as a form of intercellular communication. MVs, released in the culture medium by NSCs during development, were confirmed to play a protective role in nerve damage, modulate neuronal activity, and play an important role in the development and function of the nervous system [9, 14, 15]. These genetic information in MVs from stem cells were confirmed to repair damaged tissues without directly replacing cells [16]. It has been demonstrated that there were a negative correlation between lesion volumes and NSC-derived MVs in the ischemic stroke patients [17], which suggested that transplantation of NSC-derived MVs could decrease cerebral infarct volume. In addition, a previous study also demonstrated that MVs from human NSCs were able to attenuate neuroinflammation and preserve host neuronal morphology in the irradiated brain [18]. In addition, transplantation of NSC-CM into injured mouse brains not only caused expansion of the NSC population in the subventricular zone but also enhanced the formation of new neurons that migrated to the damaged site [19]. NSC-CM was confirmed to have the capacity of inducing mesenchymal stem cells into neural stem cell-like cells *in vitro* [20], which further suggested that NSC-CM might enhance endoneurogenesis. More importantly, NSC-CM could significantly attenuate neuron apoptosis after spinal cord injury in rats [21]. Our previous study also suggested that NSC-CM significantly inhibited cell apoptosis and enhanced neuronal differentiation of RA-differentiated SH-SY5Y cells *in vitro* [22]. Therefore, we hypothesized that NSC-CM might have the capacity of protecting against cerebral I/R injury.

Thus, in the current study, we tested whether intravenously injected NSC-CM could improve the neurological functional recovery and reduction of cerebral infarction volume caused by cerebral ischemia/reperfusion (I/R) injury in rats. Furthermore, we tried to investigate the possible neuroprotective mechanisms of NSC-CM on cerebral I/R injury in rats.

## 2. Materials and Methods

### 2.1. NSC-CM (Neural Stem Cell-Conditioned Medium) Preparation

All animal protocols and procedures in the current study were reviewed and approved by the guidelines of the Ethical Committee for Animal Experiments of Shandong University. E15–18 pregnant Sprague-Dawley (SD) rats were purchased from the Animal Research Center of Shandong Traditional Medicine University (Jinan, China) and used for isolating neural stem cells (NSCs). The method of isolating and culturing NSCs was performed according to the protocol by Kim et al. [23], and the detailed method of collecting NSC-CM was performed according to the protocol we previously provided [22]. In brief, the cortex region of the E15–18 SD rats was isolated, and the meninges was peeled off on the clean bench. The cortexes were transferred to a 15 ml conical tube containing 3 ml HBSS (Hanks balance salt solution) for 5mins, then dissociated into small pieces using a 1 ml pipette tip. 3 ml HBSS containing small pieces of cortexes was filtered by 100 nm filters and centrifuged at 1000 RPM∗10 minutes at RT (room temperature) to get single cells. After that, the cells were resuspend in a completed culture medium including DMEM/F12 (Invitrogen, CA, USA), human recombinant epidermal growth factor (EGF; 20 ng/ml) and basic fibroblast growth factor (bFGF; 20 ng/ml) (R&D Systems, Minneapolis, MN, USA), B27 (serum-free medium supplements formulated to provide optimal growth condition for NSC expansion, 1 : 50; Invitrogen), heparin (5 *μ*g/ml; Sigma, St Louis, MO, USA), 2 mM L-glutamine, and an antibiotic-antimycotic mixture (1 : 100; Invitrogen, 10,000 u/ml penicillin, 10,000 *μ*g/ml streptomycin, and 25 *μ*g/ml amphotericin B). The number of viable cells in the suspension was assessed using trypan blue, and cell density of the suspension was adjusted to 2∗10^5^ cells/ml. Then, the cell suspensions were seeded into a T-75 flask containing a 15 ml completed culture medium at 37°C in a 5% CO_2_-humidified incubation chamber (Fisher, Pittsburgh, PA, USA) for 4days. After 4days, the single cells were cloned into NSC spheroids, and the medium was changed completely with a fresh completed culture medium. A 7.5 ml fresh completed culture medium was changed every 3 days. Every 2 weeks, we cut the larger neurospheroids into small spheroids under the observation of the microscope (Olympus, Japan). At the time of each medium change, we collected the rat NSC conditioned medium by filtering through a membrane with a pore size of 0.4 *μ*m in diameter (Millipore, Billerica, MA, USA). The filtered conditioned mediums were centrifuged at 1000 RPM∗10 minutes at RT. After that, we observed the medium under the microscope to make sure that there was no cell contamination. The neural stem cell-conditioned medium was kept at 4°C for 7 days.

### 2.2. Animal Model Preparation and Treatment

Male Sprague-Dawley rats (*n* = 40) weighing 150–200 g were purchased from the Animal Research Center of Shandong Traditional Medicine University (Jinan, China). The animals were kept under standard laboratory conditions, maintained in temperature and humidity controlled rooms on a 12 h/12 h light/dark cycle, and had free access to food and water. The cerebral ischemia/reperfusion (I/R) injury induced middle cerebral artery occlusion (MCAO) as previously described by our group [24]. Briefly, the rats were anaesthetized by 10% of chloral hydrate (3 ml/kg BW, i.p.) and the right carotid bifurcation, the right common carotid artery (CCA), the right internal carotid artery (ICA), and the right external carotid artery (ECA) were exposed to the performer by a ventral neck incision. The monofilament nylon with a silicone-beaded tip (Sunbio Biotech, Beijing), 0.28 mm in diameter, was labeled at 18 mm to the silicone-beaded tip before inserting into the right ICA. The monofilament nylon was inserted into the right ICA until resistance was felt at 16–20 mm from the bifurcation of the right CCA. The monofilament nylon was then fixed and carefully withdrawn after 90 min of middle cerebral artery occlusion to permit reperfusion. Throughout the procedure, body temperature was maintained at 37 ± 0.5°C with a thermostatically controlled infrared lamp. The cerebral ischemia/reperfusion rats were randomly divided into 2 groups. One group was slowly administrated with 1.5 ml NSC-CM by tail vein injection at 3 h, 24 h, and 48 h after ischemia onset. The other group was slowly administrated with 1.5 ml PBS (phosphate buffer saline) by tail vein injection at 3 h, 24 h, and 48 h after ischemia onset.

### 2.3. Neurological Defect Score and Cerebral Infarct Volume Measurement

The blind examiner assessed the neurological defect scores at the 3rd day after ischemia onset. The 21-point behavioral scale (normal and maximum score, 21) was used to evaluate neurologic defects according to the previous report [25]. A lower score correlated with the worst neurological defects. Data of 10 rats from each group were averaged, expressed as the mean ± SEM, and compared between two groups.

After assessment of the neurological defect score, rats (*n* = 4 per group) were killed using overdose of 10% of chloral hydrate (4 ml/kg BW, i.p.) for the isolation of the brains to measure cerebral infarct volumes using TTC (2, 3, 5-triphenyltetrazolium chloride) staining as previously described by us [24]. The isolated brains were stored at −20°C for 20 min; then, five coronal sections were dissected and incubated in 2% TTC (Sigma, USA) at 37°C for 30 min. After incubation, all sections were fixed in 4% PFA (paraformaldehyde buffer) for 24 h; the IPP6.0 system (Media Cybernetics, USA) was used to calculate the cerebral ischemic volume. The total ischemic volume was expressed as a percentage of cerebral ischemic volume in the hemisphere ipsilateral to the lesion. Data from 4 rats from each group were averaged, expressed as the mean ± SEM, and compared between two groups.

### 2.4. TUNEL Staining

After assessment of the neurological defect score, rats (*n* = 5 per group) were anesthetized with 10% chloral hydrate (3 ml/kg BW, i.p.) and perfused transcardially with ice PBS followed by 4% PFA (paraformaldehyde in PBS, pH = 7.4). After that, the brains were removed and dehydrated in 30% and 20% sucrose solution. The brains were frozen in Tissuse-Tek embedding compound (Sakura Finetek, Japan) and cryosectioned on a cryostat (Leica CM1850, Germany). The sections of the brains were used for further terminal deoxynucleotidyl transferase-mediated dUTP nick and labeling (TUNEL) staining. TUNEL staining (Boster, Wuhan, China) was performed to detect apoptotic cells in the ischemic hemisphere and applied according to the manufacturers' instructions. In brief, five serial sections with an interval of 50 *μ*m were randomly obtained from each rat. After incubating in 0.025% 3, 3-diaminobenzidine (DAB, Boster, Wuhan, China) plus 0.033% H_2_O_2_ in PBS for 10 min, the sections were counterstained with hematoxylin. After that, the sections were dehydrated, covered with neutral balsam, and examined with a light microscope (Olympus, Japan). IPP6.0 was supplied to calculate TUNEL staining-positive cells. Five regions within the cortex and penumbra per section were randomly selected for cell counting on the cerebral ischemia hemisphere at 20 × magnification. The total cell numbers and TUNEL-positive cells were obtained in each region. The percentage of TUNEL-positive cells is described as the percentage of the numbers of TUNEL-positive cells to the total numbers of cells in each region. Data from five regions of ten sections were averaged, expressed as the mean ± SEM, and compared between two groups.

### 2.5. Western Blot Analysis

After assessment of neurological defect score, rats (*n* = 5 per group) were killed by overdose of 10% chloral hydrate (4 ml/kg BW, i.p.) and the ischemic hemisphere was isolated for further Western blot analysis. Each ischemic hemisphere was centrifuged at 12,000 rpm for 30 minutes at 4°C. The supernatant was collected, and protein concentration was measured using a BCA protein assay kit (Beyotime, Shanghai, China). Protein extract and sample buffer were mixed and boiled 5 minutes at 100°C before loading onto 15% polyacrylamide gels. We performed Western blot analysis using standard techniques with an ECL Plus detection kit (Millipore, Billerica, MA). The antibodies used in Western blot analysis were rabbit anti-Bcl-2 (Boster, Wuhan, China, 1 : 200) and mouse *β*-actin (1 : 1000; ZSGB-Bio). Every sample was repeated 3 times for Western blot analysis. Bands were normalized to *β*-actin levels, and the density of the band was measured using ImageJ analysis software (NIH, Bethesda, MD). Data were averaged, expressed as the mean 6 SEM, and compared between 2 groups.

### 2.6. Electron Microscopy

After assessment of the neurological defect score, rats (*n* = 4 per group) were killed by overdose of 10% chloral hydrate (4 ml/kg BW, i.p.) and rapidly isolated 1 mm^3^ ischemic hemisphere cortex for the next electron microscopy analysis. The above isolated ischemic hemisphere cortexes were immediately postfixed in 3% glutaraldehyde (3% in 0.1 M cacodylate buffer, pH = 7.4) at 4°C overnight. Following rinsing three times with PBS, the ischemic hemisphere cortexes were then osmicated in 1% osmium tetroxide in PBS for 2 h, dehydrated in increasing concentrations of ethanol (30%, 50%, 70%, 80%, 90%, 95%, and 100%) for 15 min and embedded in Epon 812 resin. Ultrathin sections (0.06 *μ*m) were sliced and stained with uranyl acetate and lead citrate, then examined with JEM-1200 EX electron microscope by a blind examiner.

### 2.7. Statistical Analysis

Statistical analysis was performed using GraphPad Prism 5 (GraphPad Software, La Jolla, CA). Data were expressed as means with SEM. Two-sample *t* test was used for the data analysis. Significance was set at *P* < 0.05.

## 3. Results

### 3.1. NSC-CM Significantly Protected against the Neurological Defect Caused by Cerebral Ischemia/Reperfusion Injury

**Figure 1:**
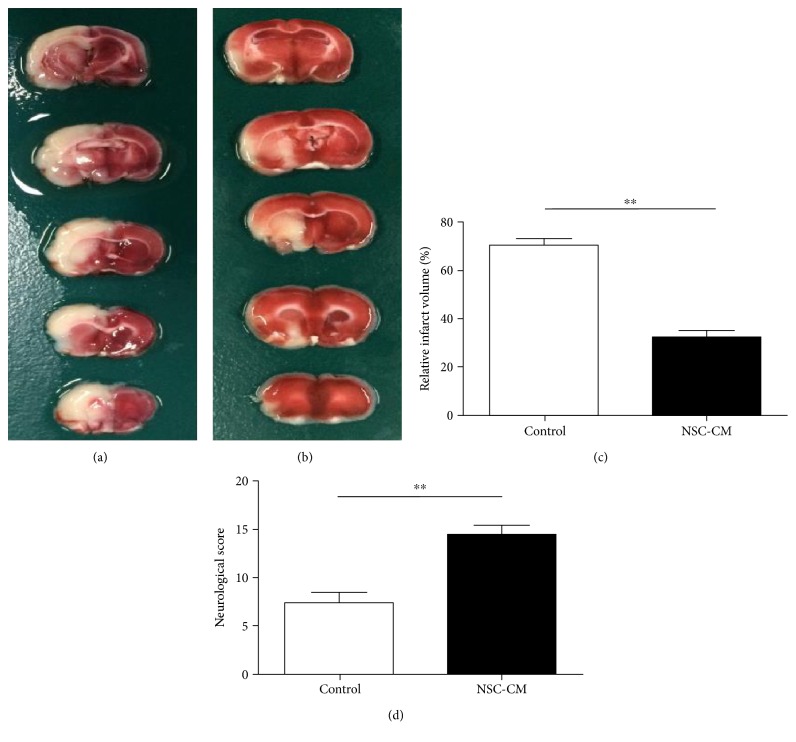
The effect of NSC-CM on the neurological defect score and cerebral infarct volume of cerebral ischemia/reperfusion injury. Representative TTC staining image on the 3rd day after cerebral ischemia onset of the control group (a) and the NSC-CM group (b). The bar graph showing that the percentage of cerebral ischemic volume (c) in the hemisphere ipsilateral to the lesion on the 3rd day after cerebral ischemia onset was significantly decreased by posttreatment with NSC-CM compared with the control group (*n* = 5 each group). ^∗∗^*P* < 0.01, two-sample *t* test. The bar graph showing that NSC-CM (d) significantly improved the neurological defect score according to a 21-score point, compared to control. ^∗∗^*P* < 0.01, two-sample *t* test.

To evaluate whether NSC-CM exert neuroprotective effect on cerebral I/R injury in rats, we measured the neurological defect scores and cerebral infarct volumes on the 3rd day after cerebral ischemia onset. We applied a 21-point scale to assess the neurological defect scores of rats on the 3rd day after cerebral ischemia onset. A lower score correlated with the worst neurological defects. As demonstrated in Figure 1(d), we found that the neurological defect scores of rats receiving NSC-CM tail vein injection were significantly higher than the control group (*P* < 0.01). In addition, we also performed TTC staining to measure the cerebral infarct volumes. As illustrated in Figures 1(a), 1(b), and 1(c), we observed that NSC-CM vein tail injection significantly reduced the cerebral infarct volumes, compared to control group (*P* < 0.01). Thus, these data indicated that NSC-CM had the capacity of protecting the rats against cerebral I/R injury.

### 3.2. NSC-CM Significantly Attenuated Cell Apoptosis via Improving the Expression of Bcl-2

**Figure 2:**
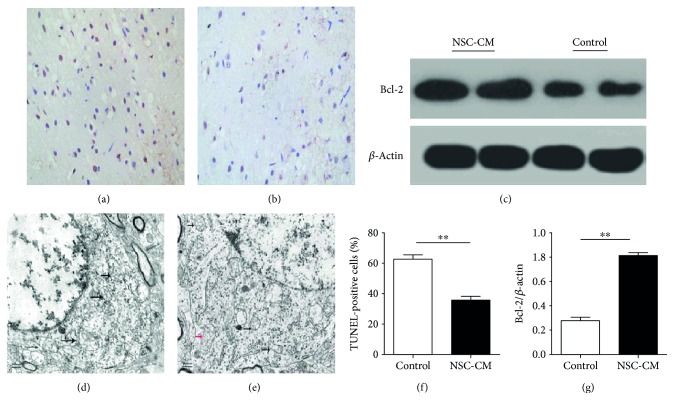
The effect of NSC-CM on cell apoptosis and mitochondrial ultrastructure in the ischemic sphere. Representative TUNEL staining image on the 3rd day after cerebral ischemia onset of the control group (a) and the NSC-CM group (b). The brown particle is the TUNEL-positive cells whereas the blue particle is the normal cells (c). The protein expression of Bcl-2 in the cerebral ischemia penumbra of 2 groups assessed by Western blot. Control: the group receiving PBS by tail vein injection; NSC-CM: the group receiving NSC-CM by tail vein injection. Representative mitochondrial ultrastructures of the control group (d) and the NSC-CM group (e) in neuron. The black arrows indicated swollen mitochondria with disintegrated or disappeared cristase. The red arrows indicated normal mitochondria with integrated cristase. Bar: 500 nm (f). The bar graph showing that NSC-CM significantly decreased the number of TUNEL-positive cells in cerebral ischemic hemisphere, compared to the control group. ^∗∗^*P* < 0.01, two-sample *t* test (g). The bar graph showing that NSC-CM significantly increased the expression of Bcl-2 in cerebral ischemic hemisphere, compared to control group. ^∗∗^*P* < 0.01, Two-sample *t* test.

To investigate the possible mechanisms of the neuroprotective effects of NSC-CM on cerebral I/R injury in rats, we applied TUNEL staining to measure the number of apoptosis cells in the ischemic hemisphere. As demonstrated in Figures 2(a), 2(b), and 2(f), NSC-CM significantly reduced the number of apoptotic cells in the ischemic hemisphere, compared to the control group. To further clarify how NSC-CM inhibit cell apoptosis, we applied Western blot to measure the expression of Bcl-2 in the cerebral hemisphere. As illustrated in Figures 2(c) and 2(g), NSC-CM significantly increased the expression of Bcl-2 in the ischemic hemisphere (*P* < 0.01). Thus, these data suggested that the capacity of NSC-CM to improve the expression of Bcl-2 in the ischemic hemisphere might contribute to inhibit cell apoptosis.

### 3.3. NCS-CM Significantly Preserved Mitochondrial Ultrastructure in the Cerebral Ischemia Hemisphere

To further clarify whether NSC-CM have the capacity of preserving mitochondria, we used electron microscopy to observe the mitochondrial ultrastructure. As illustrated in Figure 2(d), the mitochondria in the cerebral I/R injury rats became swollen. Especially, mitochondrial cristae almost disappeared or appeared disintegrated. But, in the NSC-CM tail vein injection group, we still observed some normal mitochondria with integrated cristae although most mitochondria became swollen and appeared disintegrated cristae (Figure 2(e)). More importantly, the mitochondria in the NSC-CM tail vein injection group were less severely swollen than the control group. Thus, the above data indicated that preserved mitochondrial ultrastructure caused by NSC-CM contributed to neuroprotection against cerebral I/R injury.

## 4. Discussion

Despite the promising effect of using NSCs to improve the recovery of cerebral I/R injury, issues like ethics, immunorejection, potential tumor formation, and low survival and differentiation rates in the affected brain still limit the clinical application of NSCs [5]. NSC-CM is always discarded as the waste because NSCs produce possible harmful materials in NSC-CM during cell division *in vitro*. But, NSC-CM has been receiving more and more attentions in recent years because bystander actions of NSCs in vivo are extensively investigated, especially MVs released by NSCs. NSC-CM was demonstrated to exert antiapoptotic effect in vitro [22] and in vivo [21]. To circumvent some potential confounders and find a novel therapeutic strategy for ameliorating cerebral I/R injury, we for the first time explored the effects of multiple tail vein injections of NSC-CM in a cerebral I/R rat model and found that NSC-CM effectively ameliorated cerebral I/R injury in rats.

As expected, our data suggested that continuous administration of NSC-CM significantly reduced the cerebral infarct volume and improved the neurological defect scores (Figures 1(b) and 1(c)). Ischemic stroke is considered to be caused by decreased blood flow to brain tissue, followed by the activation of the ischemic cascade which leads to cell death and severe neuronal damage [26], which includes apoptosis, oxidative stress, inflammation, and calcium overload. It has been demonstrated that apoptosis plays an important role in cerebral ischemic pathogenesis in rats [27]. NSC-CM include neurotrophic factors, like GDNF and BDNF, while BDNF or GDNF was confirmed to exert antiapoptotic effect after transient middle cerebral artery occlusions in rats [28, 29]. In addition, NSC-CM was also confirmed to inhibit the cell apoptosis of SH-SY5Y cell caused by RA (retinoic acid) in vitro [22]. Thus, to dissect the potential mechanisms underlying these beneficial neuroprotective effects of NSC-CM, we performed TUNEL staining to observe cell apoptosis. Consistent with the above two researches [22, 29], NSC-CM significantly reduced the number of TUNEL-postive cells in the ischemic hemisphere (Figures 2(a), 2(b), and 2(f)), which indicated that NSC-CM had the capacity of inhibiting cell apoptosis in vivo.

It is not new that Bcl-2 has a protective role after focal ischemia [30], and Bcl-2, as the one of antiapoptotic proteins, was one of the reduced genes most characterized after focal ischemia [30]. In addition, it also has been demonstrated that BDNF was able to protect against cerebral I/R injury via improving the expression of Bcl-2 [28]. MVs secreted by NSCs were confirmed to have the capacity of attending the process of inflammation [18] while inflammation contributed to the cell apoptosis in the cerebral I/R injury [1]. Thus, we guessed that NSC-CM had the capacity of inhibiting cell apoptosis. To further explore the mechanisms of NSC-CM against apoptosis, we selected Bcl-2 as the key antiapoptotic protein. Our data suggested that NSC-CM significantly improved the expression of Bcl-2 in the ischemic hemisphere (Figures 2(c) and 2(g), *P* < 0.01). More and more evidences suggested that Bcl-2 halted apoptosis by stabilizing mitochondrial integrity [27]. Mitochondria, as important components of ischemic neuronal death for several decades, is demonstrated to become swollen when cerebral I/R injury occurs [31]. More importantly, the earliest manifestations of ischemic neuronal demise were caused by the loss of mitochondrial cristae [31]. We still observed some normal mitochondria with integrated cristae although most of mitochondria became swollen and appeared disintegrated cristae in receiving the NSC-CM group (Figure 2(e)), which indicated that NSC-CM had the capacity of preserving mitochondrial ultrastructure. Thus, we concluded that preserved mitochondrial ultrastructure contributed to the increased expression of Bcl-2. It has been demonstrated that MVs secreted by NSCs play important roles in the development of the nervous system, enhancing the endoneurogenesis and inhibiting the process of inflammation [18]. It is well known that the integrity of BBB is broken during cerebral I/R injury, which is beneficial to penetrate BBB for some factors [32]. The components of NSC-CM are complicated, containing those already known neurotrophic factors, that is, BDNF [7], GDNF, and NT-3 [8], together with many other unknown soluble factors released from NSCs, as well as microparticles [9] in the CM. Although single application of BDNF, GDNF, and NT-3 can penetrate BBB and protect against cerebral I/R injury as demonstrated in previous studies by others, there are no researches about the effect of combinational application of neurotrophic factors on cerebral I/R injury. In the current preliminary studies, however, we did not focus on the detailed mechanisms of NSC-CM. Further studies are warranted to identify those soluble factors and calculate the proportions by global proteomic analysis of NSC-CM contents in the future, which may help elucidate the mechanism of NSC-CM on cerebral I/R injury. And, we in the future also will isolate microparticles using ultracentrifugation to testify their effects on cerebral I/R injury.

In conclusion, we showed that multiple tail vein injection of NSC-CM significantly ameliorated cerebral I/R injury via inhibiting cell apoptosis and preserving the mitochondrial ultrastructure. In another word, our data suggested that NSC-CM is another promising cell-free strategy for ischemic stroke.
